# Past, Present, and Future of EEG-Based BCI Applications

**DOI:** 10.3390/s22093331

**Published:** 2022-04-26

**Authors:** Kaido Värbu, Naveed Muhammad, Yar Muhammad

**Affiliations:** 1 Institute of Computer Science, University of Tartu, 51009 Tartu, Estonia; naveed.muhammad@ut.ee; 2 Department of Computing & Games, School of Computing, Engineering & Digital Technologies, Teesside University, Middlesbrough TS1 3BX, UK; yar.muhammad@tees.ac.uk

**Keywords:** brain–computer interface (BCI), electroencephalography (EEG), rehabilitation, systematic literature review

## Abstract

An electroencephalography (EEG)-based brain–computer interface (BCI) is a system that provides a pathway between the brain and external devices by interpreting EEG. EEG-based BCI applications have initially been developed for medical purposes, with the aim of facilitating the return of patients to normal life. In addition to the initial aim, EEG-based BCI applications have also gained increasing significance in the non-medical domain, improving the life of healthy people, for instance, by making it more efficient, collaborative and helping develop themselves. The objective of this review is to give a systematic overview of the literature on EEG-based BCI applications from the period of 2009 until 2019. The systematic literature review has been prepared based on three databases PubMed, Web of Science and Scopus. This review was conducted following the PRISMA model. In this review, 202 publications were selected based on specific eligibility criteria. The distribution of the research between the medical and non-medical domain has been analyzed and further categorized into fields of research within the reviewed domains. In this review, the equipment used for gathering EEG data and signal processing methods have also been reviewed. Additionally, current challenges in the field and possibilities for the future have been analyzed.

## 1. Introduction

The electroencephalography (EEG)-based brain–computer interface (BCI) is one of the most rapidly developing fields of BCI [1,2] and has potential to expand far beyond the domain of medical applications, in which they were initially most popular. The use of EEG has become possible due to the work and discovery by Hans Berger who discovered in 1924 that electrical signals of the human brain could be measured from the scalp [3]. The initial discovery was made using a simple galvanometer and confirmed the possibility that neural activity could be measured by this method [4]. EEG measures the brain electrical activity caused by the flow of electric currents during synaptic excitations of neuronal dendrites and is measured via electrodes placed on the scalp [1,4]. After the initial discovery by Hans Berger, additional brainwave types and mental states associated with the brainwave types have been determined. An EEG system can provide a pathway between the brain and external device, making it possible to read biological signals and interpret certain aspects of a person’s cognitive state [5]. BCI systems are EEG systems that allow users nearly real-time control of an external actuator. EEG-based BCI applications could be used by a person to control a computer or other device using one’s thoughts without using ordinary methods of working with a computer (e.g., using hands) [6]. EEG-based BCI applications could be used also to monitor subject’s mental states such as emotions [7], concentration [8] or drowsiness [9]. BCI adds to the monitoring processes, for example, via enabling systems to be adaptive or sending alert signals.

The BCI applications have been initially developed to help people with disabilities to be able to communicate, control computers and use aiding equipment such as wheelchairs or robotic arms. One of the first BCI applications assisted individuals with speech anomalies [5]. The possibility of using BCI to develop prosthetic arms was considered as early as 1917 [1]. In the overview by Tariq et al., EEG-based BCI has also been used in order to develop wearable lower-limb exoskeletons, as BCI has emerged as an alternative communication system between the human brain and output devices [10]. BCI-based prosthetics are supporting patients in cases of paralysis, amputations and loss of central nervous system functionality due to other reasons [2]. BCI could also be applied in order to enhance neuroplasticity [11]. Neuroplasticity of the brain has been characterized as the capacity of the brain to change or adapt its morphology and functioning in response to experience [12]. The applications have been used in the medical domain also by people suffering from locked-in syndrome or amyotrophic lateral sclerosis (ALS) [1].

EEG-based BCI applications have been used in different areas stretching from the medical domain to non-medical domain where EEG-based BCI applications have been used for entertainment, art, as well as some other areas [5,6,13]. There have been studies concerning the non-medical domain, for example, developing devices to monitor the alertness level of employees [14]. Another aspect that has been investigated is the overall car traffic safety and avoidance of driving fatigue and drowsiness during driving that could result in fatal accidents [1]. BCI applications can be used for controlling smart homes [15,16,17,18] or a car [19,20]. In the domain of non-medical applications, the BCI could also be used for sport motor skills improvement, acting skills improvement or surgical skills improvement [11]. The applications for entertainment could include games designed for improving subject attention level or concentration level [13], but also control of drones and humanoid robots [6].

During the current review, the literature on EEG-based BCI applications in the period from 2009 to 2019 was analyzed. The current review gives an overview concerning articles and conference proceedings per year, publications per region/continent, experimental publications per year, publication distribution by domain, EEG devices used, number of EEG channels used, techniques used to obtain EEG data, feature extraction and classification methods used.

In the first part of the review, an overview concerning the published articles as well as conference papers is also presented through the years. The regions/continents at the forefront of the scientific progress in the field have been highlighted.

An overall review of EEG-based BCI applications has been created and the BCI applications have been categorized based on domain (medical or non-medical) and by field, describing the current trends in BCI applications development in more detail. The BCI applications have been included in the medical domain in case they have been designed to provide assistance, monitor, assess or support rehabilitation and in the non-medical domain in case the applications have been designed to entertain, control machines, authenticate, monitor or assist without a medical purpose. The distinction between the domains has been done in a way similar to the work by Al-Nafjan et al. [5].

This review introduces the current trends for the development of EEG-based BCI applications. Although the initial need for BCI applications has been in the medical domain, this review shows that there is currently a higher pace of development in the non-medical domain. The applications meant for widespread use could be used for monitoring user attention, supporting daily activities or for entertainment.

In this review, EEG signal acquisition and processing is also analyzed. The overview concerning the prevalence of different EEG devices has been given together with the details regarding the number of EEG channels used in previous studies. An overview is given concerning the techniques used to obtain the EEG data. Regarding the EEG signal processing, feature extraction and classification methods have been reviewed.

This review was conducted in connection to the first author’s master’s thesis [21]. This review provides a comprehensive summary of recent works conducted in the field and an overview of current obstacles that inhibit progress, both technically and in other aspects. In addition to technical aspects there could be, for example, user-related aspects that limit the use of BCI applications due to difficulty for the user to learn to use the applications and inability to generate or alter the required EEG signals.

The ethical, legal and safety considerations in BCI application development are as important as the technical aspects. The synthesis on current challenges gives a mapping of what to focus on in order to support BCI development and shows where the possible risks exist that would need to be addressed. The trends and future possibilities give a better understanding of what we could expect in years to come.

## 2. Background

BCI can be defined as a system that translates the brain activity patterns of a user into messages or commands for an interactive application [22]. A BCI is a control and/or communication system in which the user’s commands and messages are not dependent on common brain-motor periphery communication channels [23]. The BCI user’s brain activity is typically measured using EEG [22]. BCI generally functions through four distinct processes consisting of recording neuronal activity, extracting features, gathering important information and combining information for useful purposes [13]. The EEG signal analysis has been further divided into four steps which are gathering of raw EEG data, signal pre-processing, feature extraction and classification [2].

According to Padfield et al. [2], the signal analysis steps could be characterized as follows. The collected raw EEG data comprises all the EEG data collected before pre-processing and further analysis. After the collection of raw EEG data, pre-processing is used to clean noise and enhance the quality of collected EEG data for further analysis. During feature extraction, discriminative and non-redundant information is extracted from the EEG data to form a set of features. Extracted features will capture distinct signal characteristics which can be used as a basis for the differentiation between task-specific brain states. According to Padfield et al. during classification step, the task carried out by a subject is determined and corresponding actions are taken by the system. The use of specific feature extraction and classification method depends on specific type of BCI application under study.

The most common way to categorize BCI is on the basis of invasiveness. The BCI could be invasive or non-invasive, depending on whether the device is connected physically to one’s brain and the electrodes are placed inside the brain or the device will read brainwaves from the top of the scalp [1]. The use of invasive method of direct contact of the electrodes with brain is more efficient as there are less interfering factors that influence the signal quality [24], but the invasive method contains the risks of surgical procedures and related complications.

More popular and easy is the non-invasive method, where the brainwaves are read from the top of the scalp via electrodes located on the scalp. As this method also has some drawbacks, including disturbance from external noise, effects from the posture and mood of the subject, detecting a low strength signal resulting in a reduced signal quality [2], there is a significant amount of on-going research to determine the best ways for signal detection and analysis.

The most popular current non-invasive method for acquiring brainwaves for BCI is detection through EEG. The popularity of EEG is supported by the inexpensiveness of the equipment, reduced complications compared to invasive procedures, portability, easy process to set-up and use and the possibility to directly measure neural activity [1,2]. EEG reading is easy to use and has high potential to be applied by majority of the population in order to use high variety of possibilities that BCI offers. Other non-invasive methods include functional magnetic resonance imaging (fMRI), magnetoencephalography (MEG) and near-infrared spectroscopy (NIRS) which can be used separately or combined [1]. The advantages of EEG are high temporal resolution, good portability, high temporal resolution, low cost, less invasive when compared to fMRI and independence from need of a complex environment when compared to MEG according to Alsharif et al. The disadvantage of EEG is low spatial resolution [25].

BCI could be divided into separate subgroups based on the way to detect and convey the signal from the brain to BCI application and principles of the functioning of the BCI. As per Al-Nafjan et al. [5], BCIs could be categorized as active or passive, based on the control of the BCI application. The categorization of BCIs based on active or passive control and corresponding techniques used to obtain EEG data is presented in Table 1.

The BCIs using active control react on conscious efforts to alter brainwave patterns and the applications could be controlled via active efforts by the user. The BCIs applying passive control react to the involuntary status of the brainwaves, for example on emotional states such as meditation, excitement and stress. Different emotions could be elicited by, for example, visual-based elicitation using images, prepared task or audio-visual elicitation using short film video clips [5].

During active control of the BCI application, the signal could be detected via different techniques. The range of these techniques is broad and covers motor imagery, external stimulation (such as visual, auditory and vibrotactile), error-related potential, hybrid and other techniques. During the motor imagery task, for example, the subject is imagining the movement of a specific body part, and during error-related potential task, error-related potential is generated when there is a mismatch between the subject’s intentions and response from the BCI application [1].

As per Pasqualotto et al. [23] and Machado et al. [26] BCI could also be categorized depending on whether BCI is dependent or independent of certain muscle movements. Padfield et al. [2] have also categorized BCI as evoked or spontaneous. As per Nicolas-Alonso and Gomez-Gil [27] BCI could be categorized as synchronous or asynchronous. An overview concerning additional different categorization of BCIs in the literature is presented in Table 2.

“Dependent BCIs” require muscle control, for example, via gaze control. “Independent BCI” on the other hand detect signals only based on changes in the brainwaves without required muscle movement [23,26]. According to Padfield et al. [2], another possibility to categorize BCIs would be depending on whether external stimulation is required for the functioning of the BCI or not, thus dividing the systems into “evoked” when external stimulation is needed and to “spontaneous” in case external stimulation is not needed. According to the aforementioned categorization by Padfield et al., evoked systems include for example steady-state visual evoked potential (SSVEP), where visual stimulation is received via flickering at unique frequencies that causes corresponding changes in EEG when focusing on specific stimulus at a specific flickering frequency. According to this categorization spontaneous systems also include for example motor-imagery technique, where external stimulation is not needed and the changes in EEG patterns are generated via imagining the movement of a limb. Padfield et al. have noted that the categorization to evoked and spontaneous systems has also been named by some authors as exogenous and endogenous.

An additional way to classify BCIs is based on the time when the signals from the user are gathered by the BCI. The BCIs could therefore be divided as synchronous and asynchronous [27]. BCI is considered synchronous, when the information concerning the brainwave status is gathered during specific time intervals. It means that the user can give commands only at distinct timing, and the brainwaves are not measured at other times. In case of asynchronous analysis, the brain patterns are analyzed on an ongoing basis and the user is more flexible when giving commands to the system.

## 3. Objectives

The systematic literature review was prepared based on three databases, PubMed, Web of Science and Scopus, in order to cover a wide range of reliable peer reviewed publications on EEG-based BCI applications. In previous studies, either one [5] or multiple databases [28,29] have been used to conduct the literature search, but in the current review a selection of multiple databases was used in order to cover a broader range of publications on the topic.

The time period for the publications from between 2009 and 2019 was selected in order to give an overview concerning longer period of time and to be able to compare the results with other studies. While 2019 might not seem truly up to date, we would like to highlight that it is a systematic review. Thus, once such a review has begun, altering the chosen period would mean either (i) compromising the systematic nature of the review, or (ii) conducting a new systematic review from scratch, which are undesirable and impractical, respectively. Similar lengths of the time interval have been selected in previous studies [5,28]. From the time period above, 635 publications were selected according to the search criteria specified in Appendix A, which were then further screened and assessed for eligibility. As this review includes articles until 2019, in Appendix B we list additional publications which have been published after the time period of the current review, i.e., 2009 to 2019.

After the identification of the publications, a screening of the publications was performed. After the assessment of eligibility, 202 publications were included in the final analysis. The process was conducted as per the PRISMA model [30]. Listed below are the objectives that were set at the beginning for conducting our systematic literature review.

The objectives of the systematic literature review on EEG-based BCI applications:

1. Determine the trends concerning the publication of articles, conference proceedings and overall number of publications on EEG-based BCI applications from 2009 until 2019.
2. Determine the techniques used for obtaining EEG data and give an overview concerning the trends in EEG signal processing, which includes feature extraction and classification methods.
3. Give an overview concerning the devices used for EEG signal collection and specify the number of EEG channels used for obtaining the data.
4. Determine the proportion of scientific studies conducted on the topic in the medical and non-medical domain and further analyze the distribution of the studies per application field.
5. Analyze the literature by regions/continents, i.e., which regions/continents are at the forefront of scientific progress in EEG-based BCI applications and highlight the most contributing authors on the topic.

## 4. Methods

As per best practice, the PRISMA model [30,31] has been used to conduct systematic literature reviews in many fields of research. In addition to the PRISMA model, guidance from Cochrane Collaboration can be followed in order to prepare a systematic literature review [32]. As the PRISMA model has been developed together with the Cochrane Collaboration and with a large number of experts in the field, the PRISMA model was selected as the best current collection of principles in order to conduct the current systematic literature review on EEG-based BCI applications. The PRISMA method has been developed by Moher et al. [30] and has been used widely in conducting well organized systematic literature reviews such as [5,23,28,29].

### 4.1. Information Sources

**Figure 1:**
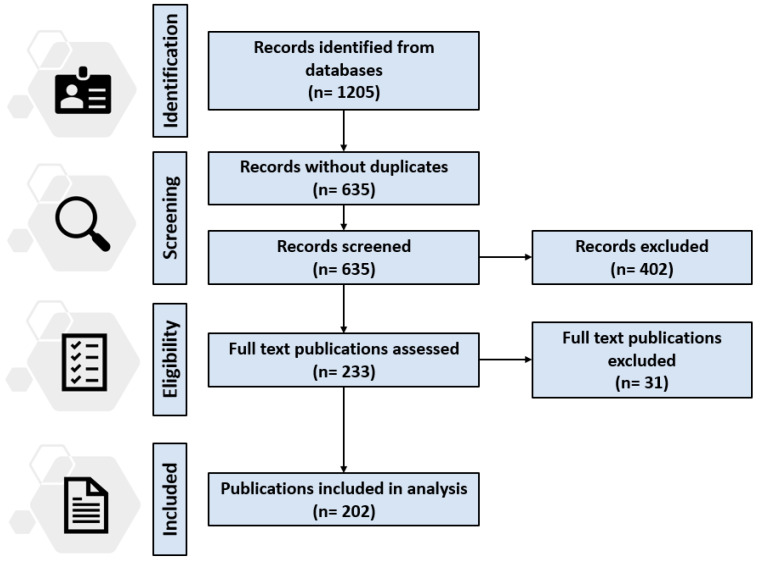
The flow of information during selection of studies according to the PRISMA model comprising Identification, Screening, Eligibility and Inclusion phase.

The publications concerning EEG-based BCI applications can be found from a high number of different sources. In the current review, three well-known databases, i.e., PubMed [33], Scopus [34] and Web of Science [35] were selected to conduct the publication search [5,28,29]. The search terms have been included in Appendix A. The process of including publications in the analysis is shown in Figure 1.

During the full review of the 202 publications included in the analysis, 25 separate data items were extracted from each publication. The extraction of the data items follows the PRISMA model for preparing systematic literature reviews, and categorization of the extracted data items has also been applied in the past by Roy et al. [28] in a similar way while following the PRISMA model.

### 4.2. Eligibility Criteria

Selection of the publications for the current review was based on pre-determined eligibility criteria. The eligibility criteria were determined to filter relevant publications concerning EEG-based BCI applications as per objectives of this review for further analysis. The eligibility criteria were selected according to similar principles used in previous review studies and studies that follow the PRISMA model [5,23,28,29,30].

The following eligibility criteria were applied during review of the publications during screening and eligibility assessment:

- Publications needed to be relatively current. In the further analysis, publications were included from the period between 2009 and 2019.
- We excluded meeting abstracts, book chapters, masters and doctoral dissertations and non-English publications.
- We excluded non-peer-reviewed journal articles and non-peer-reviewed conference proceedings.

Following the above-mentioned inclusion and exclusion steps, a manual scanning was conducted for the titles, keywords and abstracts of the publications. The screening process was conducted by one person. Publications that did not address the subject, but rather mentioned the subject in passing in general were excluded. During the review, 402 publications were excluded that did not meet the eligibility criteria. The scanning resulted in 233 publications for further full text assessment of eligibility. The overview concerning the process of identification, screening, assessing the eligibility and inclusion of the publications in the final analysis as per PRISMA model is shown in Figure 1.

During the full text review of 233 publications, 31 publications which did not correspond to the eligibility criteria were further excluded. After all steps of publication selection per PRISMA model were completed, 202 publications were included (i.e., left at the completion of the process) for the final analysis, which are [1,2,3,4,5,6,7,8,9,10,11,13,14,15,16,17,18,19,20,24,26,36,37,38,39,40,41,42,43,44,45,46,47,48,49,50,51,52,53,54,55,56,57,58,59,60,61,62,63,64,65,66,67,68,69,70,71,72,73,74,75,76,77,78,79,80,81,82,83,84,85,86,87,88,89,90,91,92,93,94,95,96,97,98,99,100,101,102,103,104,105,106,107,108,109,110,111,112,113,114,115,116,117,118,119,120,121,122,123,124,125,126,127,128,129,130,131,132,133,134,135,136,137,138,139,140,141,142,143,144,145,146,147,148,149,150,151,152,153,154,155,156,157,158,159,160,161,162,163,164,165,166,167,168,169,170,171,172,173,174,175,176,177,178,179,180,181,182,183,184,185,186,187,188,189,190,191,192,193,194,195,196,197,198,199,200,201,202,203,204,205,206,207,208,209,210,211,212,213,214,215,216].

## 5. Results

The results have been divided into Section 5.1, Section 5.2, Section 5.3, Section 5.4, Section 5.5, Section 5.6, Section 5.7, Section 5.8 and Section 5.9. The first part of the results covers the distribution of articles and conference proceedings per year, publications by regions/continents, experimental publications per year and publication distribution by domain. The sections in the second part focus on the EEG devices used, number of EEG channels, signal analysis, techniques used to obtain EEG data, feature extraction and classification. The results reflect the publications matching the search terms used during the Identification phase.

### 5.1. Articles and Conference Proceedings per Year

**Figure 2:**
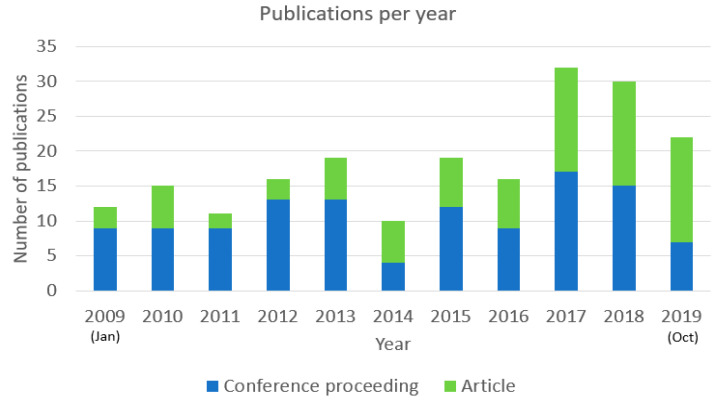
The number of publications (conference proceedings and articles) per year from the period from January 2009 to October 2019.

During the period from 2009 to 2019, the overall number of articles and conference proceedings published per year had been gradually rising. In the beginning of the period, the total number of the publications had been 11–15 publications per year from 2009 until 2011. During the period from 2012 to 2016, there was a slight increase in the volume of the publications to 16–19 publications, and only in year 2014 was there a decrease to 10 publications per year. Significant increases can be noted from year 2017 onwards when the number of publications per year had increased to up to 32 publications per year in 2017. Please see the overview concerning the number of articles and conference proceedings per year in Figure 2. As the search of the publications was conducted in the three databases during the period from 20 October 2019 to 30 October 2019, the final number of publications for 2019 could be a slightly higher number.

The trend concerning increase in the overall number of publications per year has also been noted by Al-Nafjan et al. [5] when analyzing the volume of EEG-based emotion recognition publications. In the aforementioned article, there has been a rapid increase in the overall number of publications on the topic from 2010 to 2016, and it was suggested that the increase could be caused by increased knowledge of neurobiological processes, computers with faster computational processing, greater availability of devices for recording brain signals and more powerful signal processing and machine learning algorithms. Increase in the number of publications concerning EEG-based BCIs has been also demonstrated by Hwang et al. [99], where a significant increase has been illustrated during the period from 2007 to 2011.

It can be noted from Figure 2 that during the period from 2009 to 2019, the overall number of conference proceedings published on EEG-based BCI applications has been higher than the number of articles published. Out of the 202 publications, 117 are conference proceedings (58% of the publications) and 85 are articles (42% of the publications). Although in the years from 2009 until 2016 the number of conference proceedings has been greater than the number of published articles (except for year 2014), a trend has been noted for recent years concerning the increase in the relative volume of the published articles.

The reason why the proportion of articles has increased over the recent years could be the overall development of the technology making it easier and more efficient to conduct the research on EEG-based BCI applications and inclusion of the topic more in the articles. In the beginning of the period under current focus, a higher variety and number of publications were included in conference proceedings. This trend noted in the current review illustrates the observation by Roy et al. [28] noting that there would be a wide variety of research ideas within different repositories and among different type of publications. The effect highlights the need to include a higher variety of repositories and different type of publications in the literature review in order to include objective coverage of the research ideas on the topic and avoid possible publication bias.

### 5.2. Publications per Region/Continent

The distribution of the publications concerning EEG-based BCI applications were further analyzed by region/continent. Out of the 202 publications, the highest number of publications on the topic has been published in Asia (111 publications) where the highest number of publications per country has been published in China (39 publications).

**Figure 3:**
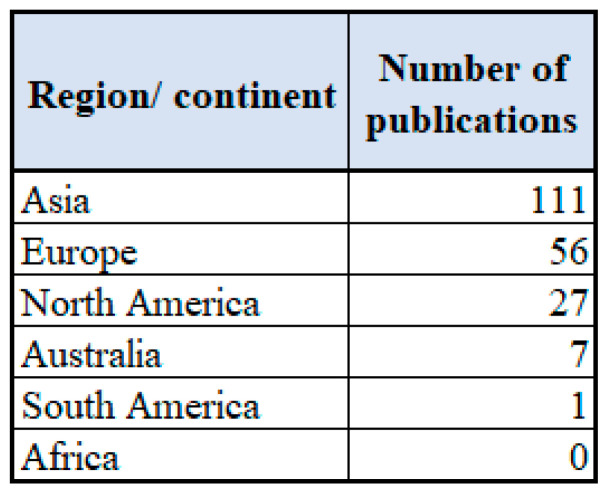
Number of publications per region/continent. The highest number of publications on the topic has been published in Asia.

The other regions/continents where higher number of publications have been published on the topic during the period of 2009 to 2019 are Europe (56 publications) and North America (27 publications). In Europe, most productive countries have been Germany and Italy (both countries with 9 publications), and in North America, the highest number of publications have been published in the USA (23 publications). Similar results have also been found by Hwang et al. [99] and Roy et al. [28] where USA and China have dominated as the countries with the highest number of publications on EEG-based BCI. A detailed overview concerning the publications per region/continent is presented in Figure 3.

Altogether, 37 different countries have contributed to the publications on EEG-based BCI applications within the time period reviewed. The highest number of contributing countries came from Europe, where the publications have been contributed by 18 different countries. From Asia, the publications have been contributed by 14 different countries, but the average number of publications per country is higher. From North America, publications have been contributed by three countries: USA, Canada and Mexico. In Australia, 7 publications were published, but from South America only one publication from Brazil has been published. It is interesting to note that no publications were contributed from Africa. It is important to note that the results reflect the publications matching the search terms used.

### 5.3. Experimental Publications per Year

Among the experimental publications on EEG-based BCI applications, non-medical publications have contributed the majority of the experimental publications per year. Exceptions to this trend have been seen in 2009 and 2013 when more medical publications were published when compared to the number of non-medical publications per year. Although in general there has been higher number of non-medical publications per year during the period under review, during recent years the proportion of medical publications has risen, reaching up to 36% of the publications.

During recent years, the overall number of experimental publications has increased from 8–15 publications per year during 2014–2016 to 19–25 publications per year during the period of 2017–2019. The trend of a rapid increase in the overall number of publications on EEG-based BCI applications can also be seen in Hwang et al. [99].

**Figure 4:**
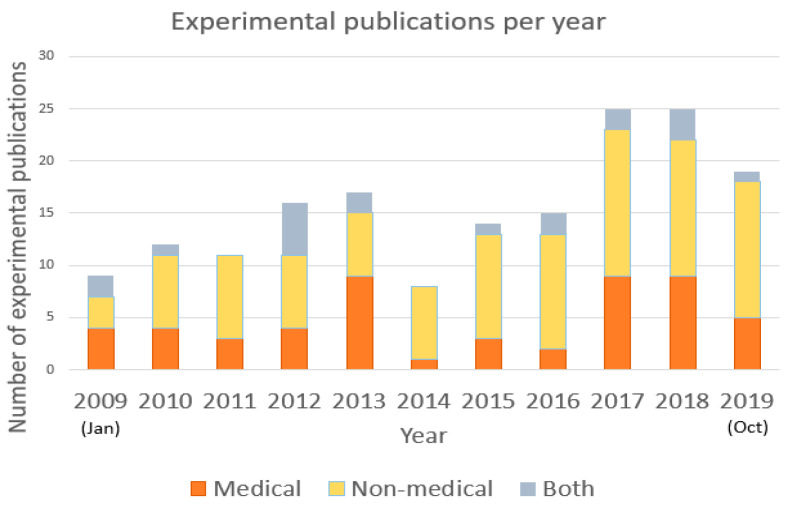
Experimental publications per year covering the period from January 2009 to October 2019. The publications have been further divided into medical or non-medical domain or both in case both domains have been covered.

Throughout the period that was analyzed, there has been 99 publications (58% of the publications) published in the non-medical domain and 53 publications (31% of the publications) in the medical domain. In 19 cases (11% of the publications) the publications cover both domains. During interpretation of the results, it should be also noted that as the database search was conducted during the period from 20 October 2019 to 30 October 2019, the final number of publications for 2019 could have been slightly increased by the end of 2019. Please see the overview concerning the number of experimental publications per year in Figure 4.

Although initially the EEG-based BCI applications were mainly developed for medical reasons to help patients to communicate, grasp objects, move around and support in other daily activities, the focus has been moving from the medical domain to non-medical applications. The shift in the focus does not reduce the importance of these applications in the medical domain but rather shows the wider potential of EEG-based BCI applications and opens doors for new possibilities.

### 5.4. Publication Distribution by Domain

**Figure 5:**
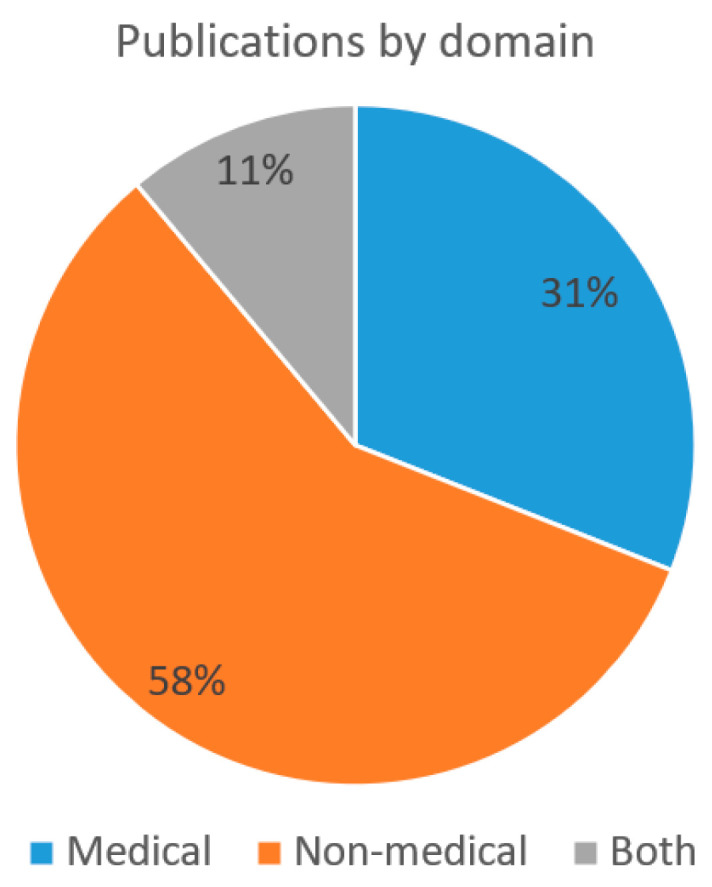
Distribution of publications per domain. The figure illustrates the distribution of the publications among medical and non-medical domain and volume of the publications covering both domains.

Among the publications within the period from 2009 to 2019, there were 58% of the publications (99 publications) from the non-medical domain. Among the overall number of publications that were analyzed, 31% of the publications (53 publications) are from the medical domain and in an additional 11% of the publications (19 publications) both domains have been included. Please see the distribution of the publications per domain in Figure 5. Similar prevalence of publications in medical domain has been determined by Al-Nafjan et al. [5], where 23% of the publications belonged to the medical domain.

The publications in the medical domain could be further divided based on the type of applications into fields such as assistive, monitoring, rehabilitation, assessment and others. Applications in the assistive field help users with disorders or disabilities to perform daily tasks and provide assistance. Within the medical domain, the field covering assistive applications is the largest contributing 74% of the publications in the domain. The assistive field includes studies on robotic arm movement in a medical setting [78], lower-limb prosthesis control [155] and intelligent wheelchair driving system [111]. The applications in the rehabilitation field for example help to restore the physical functions that have been lost by the patients due to accident or disease. Among studies analyzing possibilities for rehabilitation there is an important role for the studies supporting the rehabilitation of stroke patients [70,185,213]. As per Chaudhary et al., the assistive BCIs are intended to enable patients to communicate or control external devices and rehabilitative BCIs are intended to facilitate patient recovery [217]. When compared to assistive BCIs, the amount of rehabilitative BCIs is lower, covering 9% of the publications. An important field in the medical domain is monitoring, covering 9% of the publications in the medical domain.

The publications concerning monitoring include for example monitoring emotional changes in patients [43]. Among studies analyzing possibilities for rehabilitation there is an important role for the studies supporting the rehabilitation of stroke patients [70,185,213]. The fields of “assessment” and “other” cover a total of 8% of the publications in the medical domain, both contributing 4% of the publications in the domain.

**Figure 6:**
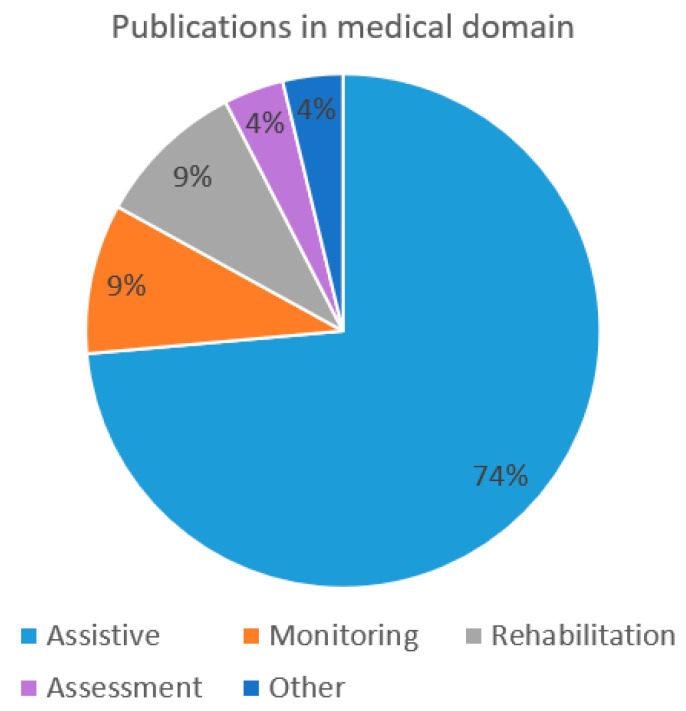
Distribution of publications in medical domain. In the medical domain, the largest field is assistive followed by monitoring, rehabilitation, assessment and other.

The prevalence of different fields in the medical domain differ when compared to previous work of Al-Nafjan et al. [5], as the most popular fields in this domain then were assessment and monitoring. The difference would be explained by the difference in the scope of the studies where in previous case the focus was on emotion recognition based on EEG-based BCI, but in the current review we focus on EEG- based BCI applications in general. An overview covering the publications in the medical domain is presented in Figure 6.

The largest field in the non-medical domain is monitoring contributing 50% of the articles in the non-medical domain. Additionally, the two large fields are control machine and entertainment contributing 17% and 16% of the publications, respectively. Smaller coverage in the non-medical domain is by the publications in the field of assistive applications covering 4% of the publications and authentication covering 2% of the publications.

**Figure 7:**
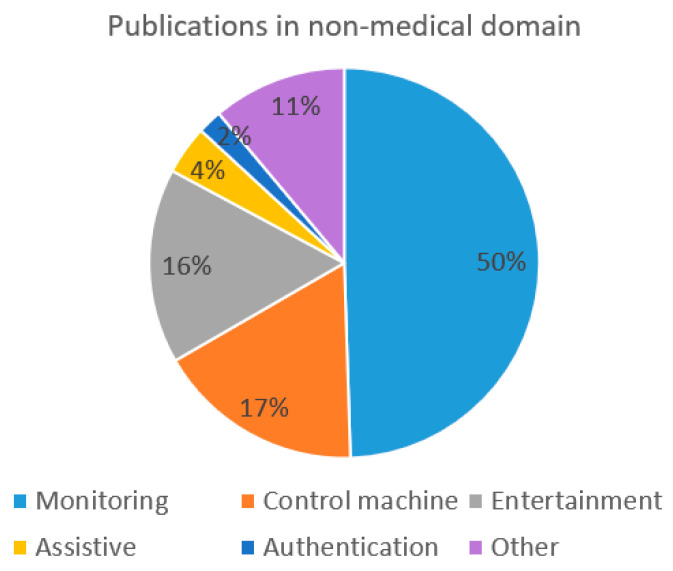
Distribution of publications in non-medical domain. In the non-medical domain, the largest field is monitoring followed by control machine, entertainment and other smaller fields.

Other different types of publications cover the rest of 11% of the publications in the domain. When compared to previous similar studies, Al-Nafjan et al. [5] has also noted that the majority of the publications in the non-medical domain concerning EEG-based BCI for emotion recognition has contributed to the monitoring field. An overview of the publications in the non-medical domain is presented in Figure 7.

In the monitoring field, a total of 49 publications have been included. The publications in the monitoring field include studies where data are collected from the subjects in order to monitor and analyze their mental activity. The studies cover for example mental fatigue estimation [182], emotion recognition [7] and detecting meditation [4]. In the control machine field, the applications include those that enable people to control different machines in their daily environment, via EEG, to make their life easier. These applications can be used for example for home appliance and smart home control [15,16] or robotic systems [38]. EEG-based BCI applications are also used for entertainment such as live brain-computer cinema performance [216], driving in a virtual city [19] or recommendations for music based on a person’s mood [174]. Other applications include user authentication using visual stimuli of geometric shapes [37], age and gender prediction [109] and other various possibilities. As illustrated in Figure 7, the majority of applications in the non-medical domain are related to monitoring. As monitoring applications have many practical applications such as monitoring mental fatigue at the workplace or during driving monitoring field has high potential for further development and practical applications for daily life.

**Figure 8:**
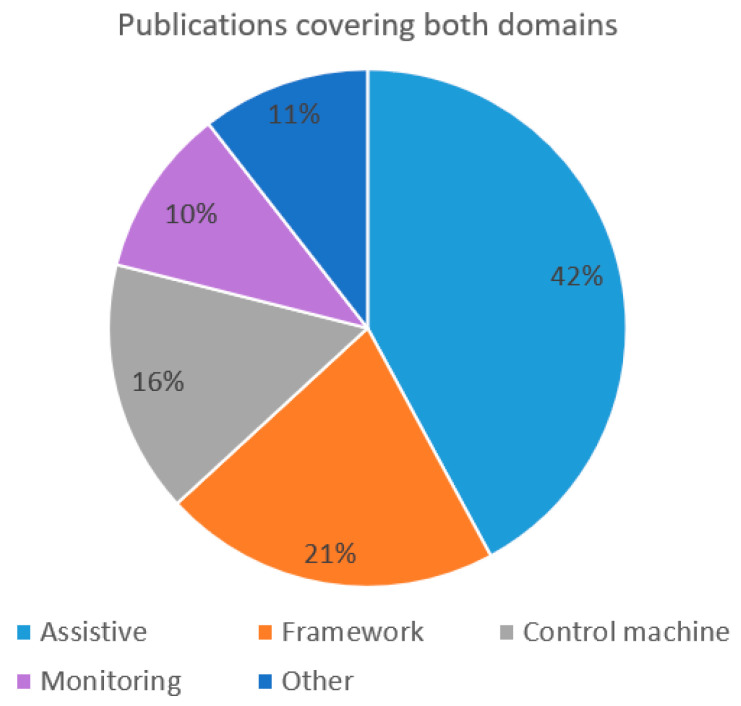
Distribution of publications covering both domains. The publications covering both domains have been focused mainly on assistive, but other fields such as framework and control machine also make up a significant proportion of the publications.

In a smaller number of the publications, both medical and non-medical domains have been covered. The domain has been further divided into the following fields: assistive, framework, control machine, monitoring and others, as per type of publications. The details concerning the distribution of publications covering both domains have been illustrated in Figure 8. Among studies covering both the medical and non-medical field, 42% of the cases publications cover the assistive category, such as detection of imagined speech and classification of unspoken words from EEG signals [85,86]. A total of 21% of the studies concern works such as developing a new framework for practical BCI communication development [57].

Studies in the field of control machine are covering 16% of the studies. They include research for example on controlling a car in an experimental environment outside laboratory conditions [20]. Some studies are focused on monitoring such as detection of kinesthetic attention [147]. Other fields cover for example the development of serious games that could be used in entertainment, e-learning or medical applications [186].

### 5.5. EEG Devices Used

Among the studies analyzing EEG-based BCI applications, the most commonly used EEG devices are Emotiv EPOC from Emotiv (San Francisco, CA, USA), Quik-Cap from Compumedics Neuroscan (Charlotte, NC, USA) and MindWave from NeuroSky (San Jose, CA, USA). These three devices combined cover 57% of the publications on the topic. The most common EEG device, the Emotiv EPOC (Emotiv, San Francisco, CA, USA), has been used in 40% of the publications analyzed while the prevalence of other popular EEG devices Quik-Cap (Compumedics Neuroscan, Charlotte, NC, USA) and MindWave (NeuroSky, San Jose, CA, USA) was 9% and 8%, respectively. The high prevalence of Emotiv EPOC (Emotiv, San Francisco, CA, USA) and Quik-Cap (Compumedics Neuroscan, Charlotte, NC, USA) use among studies analyzing EEG-based BCI applications for emotion recognition has also been noted by Al-Nafjan et al. [5] and in general for EEG equipment by Roy et al. [28].

**Figure 9:**
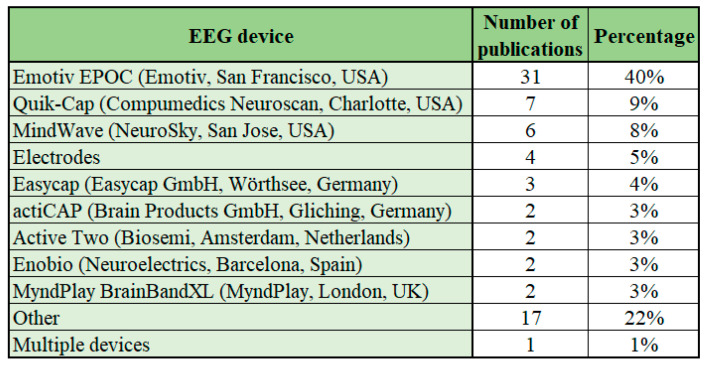
EEG devices used in the publications ordered by the number and percentage of publications where the device has been used.

The most common EEG devices (Emotiv EPOC, Emotiv, San Francisco, CA, USA; Quik-Cap, Compumedics Neuroscan, Charlotte, NC, USA; MindWave, NeuroSky, San Jose, CA, USA) are all manufactured in USA. Other EEG devices used in the studies have been manufactured also in Germany (Easycap, Easycap GmbH, Wörthsee, Germany; actiCAP, Brain Products GmbH, Gliching, Germany), Netherlands (Active Two, Biosemi, Amsterdam, The Netherlands), Spain (Enobio, Neuroelectrics, Barcelona, Spain) and UK (MyndPlay BrainBandXL, MyndPlay, London, UK). The details of different EEG devices used, number of publications and percentage of publications covered is presented in Figure 9.

Emotiv EPOC (Emotiv, San Francisco, CA, USA) and MindWave (NeuroSky, San Jose, SC, USA) are considered to be low-cost consumer EEG devices, while Quik-Cap (Compumedics Neuroscan, Charlotte, NC, USA) is more expensive to purchase [28]. While the Emotiv EPOC (Emotiv, San Francisco, CA, USA) EEG device has 14 channels and MindWave (NeuroSky, San Jose, CA, USA) has 1 channel, the Quik-Cap (Compumedics Neuroscan, Charlotte, NC, USA) EEG device uses 32 channels. The cause of the general popularity of the Emotiv EPOC (Emotiv, San Francisco, CA, USA) EEG device is due to the relative low cost of the device, sufficient number of EEG channels and the device being considered easy to use. The MindWave (NeuroSky, San Jose, CA, USA) EEG device has limitations for use due to the limited number of EEG channels, but the low cost and ease of use make the device still popular. The device could be applied in specific applications that do not require a higher number of EEG channels. The Quik-Cap (Compumedics Neuroscan, Charlotte, NC, USA) device on the other hand is more expensive but has the advantage of a higher number of EEG channels.

Dadebayev et al. [218] have found that low-cost consumer-grade EEG devices can perform equally well as research-grade devices for EEG-based emotion recognition. The advantages of the Emotiv EPOC (Emotiv, San Francisco, CA, USA) device are the high quality of the signal, less artifacts, 14 sensors, ready to use assembly and modern design. The disadvantages of the Emotiv EPOC (Emotiv, San Francisco, CA, USA) device are being non-dry sensors, 10–15 min setup time and required license for raw data access. For MindWave (NeuroSky, San Jose, CA, USA), the advantages are its low price, compactness and practical wireless device. The disadvantages of MindWave (NeuroSky, San Jose, CA, USA), as highlighted by Dadebayev et al., are the inclusion of only 1 sensor and a lower quality for EEG experiments.

The low-cost EEG headsets can be more convenient for the user and the devices such as Emotiv EPOC (Emotiv, San Francisco, CA, USA) and MindWave (NeuroSky, San Jose, CA, USA) have been successfully used in the studies developing EEG-based BCI applications [219]. The final decision concerning the use of a specific EEG device depends on the type of EEG-based BCI application determining the need for a specific number of EEG channels. The decision also depends on the cost planned for the study and devices for the end users. The EEG equipment market shows rapid development and new devices appear on the market continuously [220].

Concerning the design of the EEG devices it has been highlighted by Soufineyestani et al. [221] that the EEG headsets with dry electrodes such as MindWave (NeuroSky, San Jose, CA, USA) have been more robust to line noise when compared to EEG devices with wet electrodes, such as Emotiv EPOC (Emotiv, San Francisco, CA, USA) or Quick-Cap. According to Soufineyestani et al., the dry electrodes contain more artifacts. The dry EEG devices may lose humidity during use that may lead to decline in signal quality. In case of wet electrodes, the solution could evaporate over time, and it could be necessary to reapply the solution to the electrodes. The most convenient for the user would be dry wireless EEG devices that enable flexibility in movement and lower setup time. In case of dry electrodes, it would need to be considered that dry electrodes present lower performance when compared to wet electrodes [222]. It has been also highlighted by Soufineyestani et al. that when using wireless connectivity, the data would need to be encrypted prior to wireless transfer in order to avoid any security risk to the data of the user.

Similarly, Hinrichs et al. [223] have compared the use of dry and wet EEG systems for clinical applications and have found that although the number of artifacts was slightly higher for the dry EEG system the results were comparable between dry and wet EEG systems. The dry EEG system is more robust and less sensitive to electromagnetic interference that the subject could encounter at the clinic or at home. It has been stated by Hinrichs et al. that importantly both patients and healthy volunteers preferred the dry electrodes and reported that the dry headset was more suitable for self-application and home use.

Ratti et al. [224] have shown that although medical EEG systems offer clear advantages in data quality over consumer systems, EEG data can be collected from all systems tested in both medical and consumer contexts. Consumer EEG systems are more convenient and faster to set up. According to Ratti et al., consumer EEG systems would be more useful for a quick assessment when time is limited, while the medical grade systems would be preferred in research and clinical trial settings.

In the study by Zerafa et al. [225], a comparison was performed between a broad range of EEG devices for SSVEP BCIs. Zerafa et al. showed that low-end research grade EEG systems are comparable to the high-end research grade EEG systems in terms of signal quality. Low-cost EEG headsets suffer more from poor EEG signal quality, and it could be suggested that the best choice for developing BCI applications outside of the laboratory setting would be affordable wireless research grade systems.

When comparing the advantages and disadvantages of low-cost and more expensive EEG headsets LaRocco et al. [219] have found that traditional medical- and research-grade EEG systems have been successfully used in various applications, but are less versatile outside a laboratory environment. Low-cost EEG headsets show greater design convenience for daily occupational use and many of the low-cost devices including Emotiv EPOC (Emotiv, San Francisco, CA, USA) and MindWave (NeuroSky, San Jose, CA, USA) have been used, for example, as drowsiness detectors. LaRocco et al. have further suggested that the use of open-source software and occupational refinement may further boost the capabilities of the systems over time.

### 5.6. Number of EEG Channels

**Figure 10:**
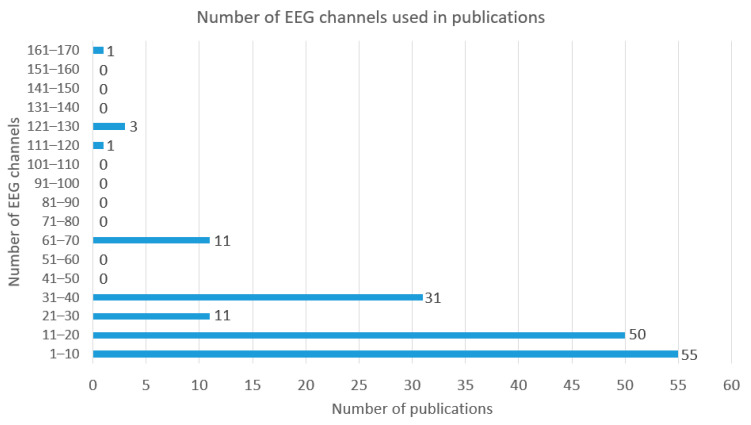
Number of EEG channels used in publications. In the majority of the publications, up to 40 EEG channels have been used.

Among the studies analyzed, the number of EEG channels used varies across a large range. The highest number of EEG channels used was 163 by Zhou et al. for EEG-based classification of elbow versus shoulder torque intentions [213]. Altogether there were 5 studies (3% of studies) where more than 100 EEG channels were used, indicating that the use of this high number of EEG channels is rather exceptional. The majority of the studies (64% of studies) involve 1–20 EEG channels, where 34% of the studies employ 1–10 EEG channels and 30% of the studies employ 11–20 EEG channels. There are also a significant number of studies that use 21–40 EEG channels and 11 studies have been conducted with 61–70 EEG channels. The detailed overview regarding the number of EEG channels used in the studies is presented in Figure 10.

Previously, Roy et al. [28] found that for obtaining EEG signals, 1 to 256 electrodes have been used, with half of the studies using between 8 to 62 electrodes. It was thought that a very high number of electrodes would not give added value to the studies, more important is the exact location of smaller number of electrodes. In that article, it was found that, concerning the number of electrodes, there is a significant increase in sensitivity and specificity when increasing the number of channels up to 22, but a further increase in the number of EEG channels would not give a similar advantage.

Al-Nafjan et al. [5] have also concluded that the majority of studies use up to 64 channels for obtaining EEG data. In the study by Al-Nafjan et al., it has been emphasized that when planning the use of a specific number of EEG channels, it is important to also consider the time required for the system setup, the comfort level for the subject and the number of features to be processed. When planning studies or developing applications for users, it is important to select the electrode positions carefully and limit the number of EEG channels. A limited number of carefully selected electrode positions would make the future devices more user friendly and optimize the system performance.

### 5.7. Technique Used to Obtain EEG Data

**Figure 11:**
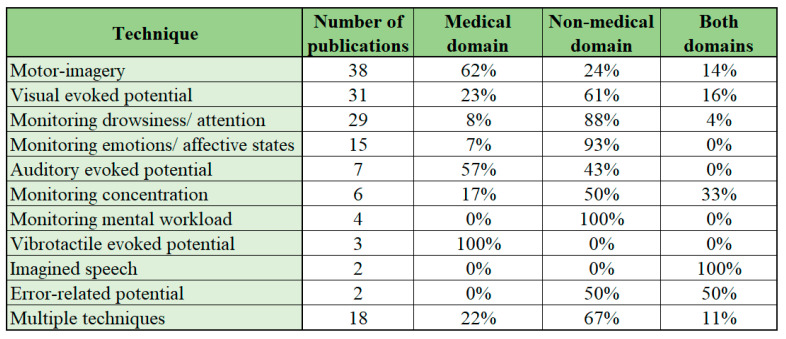
Techniques used in the publications. The techniques have been represented according to the number of publications where the techniques have been used and prevalence among medical, non-medical or both domains.

In the publications, a variety of techniques are used for the interaction between brain and computer in BCI applications. The most popular techniques include the motor-imagery paradigm (applied in 38 publications), visual evoked potential paradigm (applied in 31 publications) and monitoring drowsiness/attention (applied in 29 publications) or emotions/affective states (applied in 15 publications). The overview concerning the prevalence of different techniques is shown in Figure 11. These techniques have been used in 73% of the reviewed publications (where the technique used was stated).

Among the publications applying motor-imagery paradigm, there were 62% of the publications in the medical domain and 24% of the publications in the non-medical domain. In addition to the aforementioned, 14% of the publications covered both the domains. Among the medical domain there are innovative applications such as EConHand [163], neuro-rehabilitation using virtual reality feedback [106] and intelligent brain-controlled robotic limbs [158], applying the motor-imagery paradigm.

In the visual evoked potential paradigm, the non-medical domain is dominant as in 61% of the reviewed publications (where the technique used was stated) applied this technique. In the medical domain, the prevalence of this type of technique used was 23% and both domains were included in 16% of the cases. Visual evoked potential paradigm is widespread among BCI applications as it has been long used and tested in a high number of previous studies. The visual evoked potential paradigm also involves the visual P300 paradigm and the steady state visual evoked potential paradigm [1]. Among the most interesting applications of the aforementioned technique in the non-medical domain are the deceit identification test [74], use of EEG-based BCI devices to subliminally probe for information [83] and authentication based on emotionally significant images [166].

There is significant interest in monitoring the mind for drowsiness/attention or emotions/affective states. Both techniques use the analysis of changes in the EEG spectrum to determine the states and changes in one’s mind. The vast majority of the publications concerning drowsiness/attention (88%) or emotions/affective states (93%) are categorized in the non-medical domain. The monitoring of drowsiness/attention based on EEG spectral changes has high practical value as there are a number of studies implementing the technique in order to create helmets for on-site workers for drowsiness detection [14], predicting driver fatigue [134] or implementing EEG-based attention feedback in order to improve focus in e-learning [171].

Other techniques used in the publications are applied less frequently, but there is a general trend noted in the overall use of various techniques. As in the case of motor-imagery, auditory evoked potential and vibrotactile evoked potential technique, the main application is in the medical domain, but the majority and larger variety of techniques are applied in the non-medical domain. The trend indicates the high potential of non-medical applications for the EEG-based BCI applications. When taking into consideration that the majority of general population apply for non-medical domain, vast number of people could benefit from this type of applications in the future.

### 5.8. Feature Extraction

**Figure 12:**
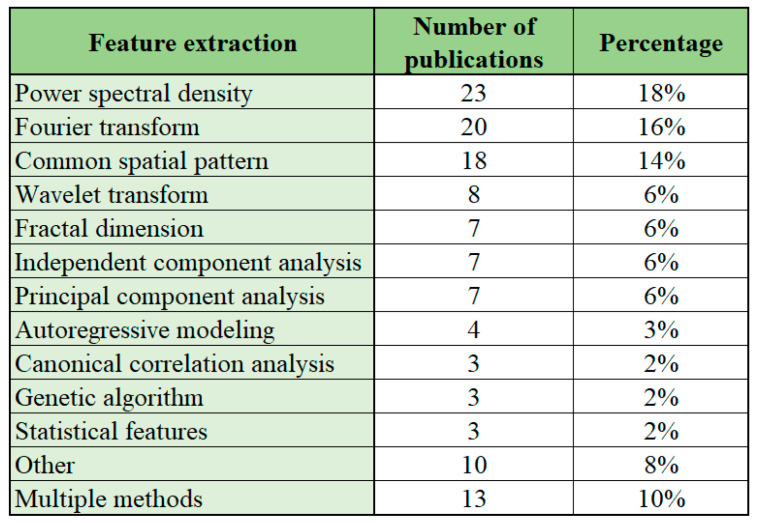
Feature extraction used in the publications ordered by the number and percentage of publications where the extraction method has been used.

The feature extraction is an important processing step in order to extract the relevant details from the wide range of signals collected during acquisition of the EEG data. In previous studies, high number of different methods have been used for feature extraction. According to Al-Nafjan et al. [5] the feature extraction step is one of the major challenges in BCI systems and the technique is not optimal across different applications. The overview concerning the prevalence of different methods for feature extraction among the studies investigating EEG-based BCI applications is presented in Figure 12.

Among the studies reviewed the most frequent feature extraction approaches are analysis of power spectral density (used in 23 publications), Fourier transform (used in 20 publications) or the analysis of common spatial pattern (used in 18 publications). Altogether, the aforementioned publications contribute to 48% of the reviewed publications. These results are similar to previous work by Al-Nafjan et al. [5], who state that power spectral density and Fourier transform have been most frequently used for feature extraction in the studies to classify emotional features from EEG.

According to Xie et al. [226] the advantage of power spectral density is feature stability. In terms of disadvantages, the power spectral density is not suitable for unstable signals, and it would not be possible to analyze time-domain signals. The Fourier transform is, according to Xie et al., applicable for stationary signals and is appropriate for narrowband signals, but does not work for nonstationary signals, greatly sustains noise sensitivity and does not have shorter duration data record. Common spatial pattern has a good effect when applied to EEG data based on motor imagination and does not require the selection of a specific frequency band in advance, but is sensitive to noise and requires multiple electrodes [226].

Other methods for feature extraction are used less frequently, but in many cases different feature extraction methods are used within one study. Similar general prevalence concerning the use of power spectral density and use of different feature extraction methods within studies has been noted by Al-Nafjan et al. [44], for analyzing human emotions from EEG, Padfield et al. [2] when analyzing EEG-based BCI interfaces using motor-imagery technique and Sourina et al. [178] for real-time brain state recognition from EEG.

Our overall results correlate with previous studies in the field highlighting the importance of analyzing power spectral density, the use of Fourier transform and common spatial pattern for feature extraction and emphasizing the need to apply different types of feature extraction methods depending on the application under study.

### 5.9. Classification

**Figure 13:**
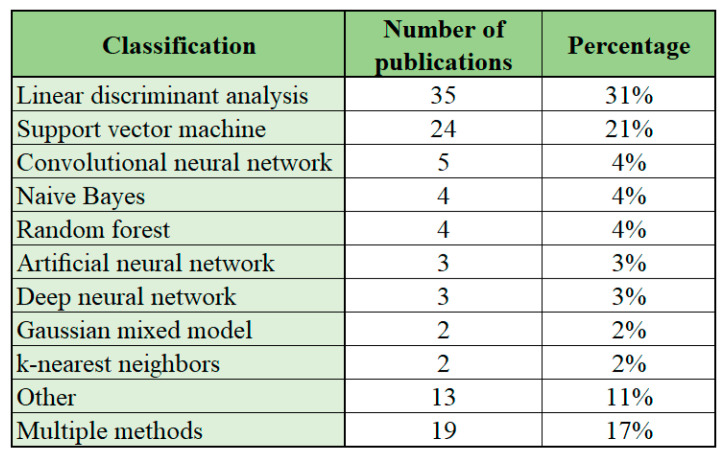
Classification method used in the publications ordered by the number and percentage of publications where the classification method has been used.

For the purpose of classification, different machine learning algorithms have been used in previous studies for EEG-based BCI applications. The most common machine learning algorithms used are linear discriminant analysis and support vector machine, which have been applied in 52% of the studies. Other machine learning algorithms have the coverage of 2–5%. Among all of the methods, linear discriminant analysis has been applied in 31% of the cases and support vector machine in 21% of the studies. An overview of the classification methods employed in the reviewed studies is presented in Figure 13.

Linear discriminant analysis has been characterized as a simple classifier with low computation requirements and acceptable accuracy [122]. Linear discriminant analysis is easy to use with low computational complexity, but requires a linear model [226]. Support vector machine is a speedy classifier that supports binary and multi-class method and can perform linear and nonlinear modes [122]. Support vector machine performance is better compared to other linear classifiers, but it has low computational complexity [226].

Other methods applied in EEG-based BCI applications are machine learning algorithms such as convolutional neural network, naive Bayes and random forest that account for 4–5% of the cases and artificial neural network, deep neural network, Gaussian mixed model and k-nearest neighbors that account for 2–3% of the cases.

The results of the current review are in correlation with previous studies where Al-Nafjan et al. [5] have shown that the use of support vector machine is one of the most popular methods used for classification for EEG-based BCI systems for emotion recognition. According to the aforementioned study, the choice for classification algorithm depends on the type of brain signal being recorded and the type of application that is being controlled. Wide use of linear discriminant analysis and support vector machine for classification has also been shown by Padfield et al. [2] for EEG-based BCIs using motor-imagery.

Dadabayev et al. [218], which pertains to feature extraction techniques and classification methods used for EEG-based emotion recognition, has stated that no feature extraction technique or classification method could be named exclusively the best option for all cases, but the right strategy relies on the specific system paradigm and objectives. It has been suggested that it would be needed to consider many different machine learning algorithms and compare the results before choosing the best model for the given objective [218]. Similarly, during a comparison of machine learning methods for emotion recognition, including linear discriminant analysis, support vector machine, naive Bayes and k-nearest neighbors, Doma and Pirouz [227] did not find that any of the methods would outperform one another.

According to Nakagome et al. [228], when comparing neural networks and machine learning algorithms for EEG gait decoding neural network-based decoders with downsampling or a wide range of frequency band features could improve the decoder performance and robustness for stable use of BCIs. Varszegi [229] has suggested that in order to improve artificial neural networks it would be further needed to look inside them, develop methods to analyze their internal activations, figure out the behavior of their architectural elements and create knowledge basis for conscious artificial neural network design to handle EEG signal processing tasks. According to Lotte et al. [22] the ideal classification method would use features and classifiers that are invariant over time, over users and contexts. There is a need for a new generation of BCI classification methods that consider the user in the loop and could adapt to the user [22].

## 6. Discussion

In the field of EEG-based BCI applications, there is a number of challenges hindering the development of the applications that would need to be addressed during future studies. It is important to acknowledge these aspects to find solutions or alternatives as needed. There are also many opportunities in the field to be used in the future and this is also an important aspect to highlight and share ideas among researchers. The sharing of new ideas and possibilities for the future facilitates the development of a large variety of applications in the field of EEG-based BCI applications.

During the preparation of this systematic review, a strict process has been followed including the use of planned search terms and databases before the analysis of the literature. The time period for the publications was selected from 2009 until 2019 for conducting of the analysis in order to give an overview concerning a longer period of time. The review was designed in order to be able to compare the results with other previously conducted reviews on similar topics. Similar time interval has been selected also in previous studies [5,28]. The additional publications published after the aforementioned time period and outside of the previous process have been included in the Appendix B of this review.

The selected search terms also set specific limitations for the publications identified and analyzed in the review. Using the term “EEG-based” could set specific limitations for the search results and term “EEG” could be used in order to obtain a wider variety of search results. The aforementioned use of specific search words could be the reason why some of the publications, for example [222,230,231,232,233], have not been identified for screening during the first phase of the review. As the publications have been identified for analysis until 2019, this aspect would need to be also considered as a limitation for the review.

### 6.1. Challenges

The challenges for the BCI applications could be divided into technology-related and user-related, where technology-related challenges comprise technical aspects and the usability of the system and the user-related contain the aspects of a person to learn to use the BCI application and subjectiveness of the person to interpret the cues that generate or alter the acquired EEG signals [5]. Padfield et al. [2] has categorized the possible challenges also as challenges faced in the research and development, challenges impeding commercialization, flawed testing process, issues with BCI use and ethical issues. The categorizations could be also combined and distinguish different technology-related and user-related challenges under the five categories proposed by Padfield et al. [2].

In terms of challenges, the major aspect would be the current reliability of the BCI system in everyday noisy environments [87]. This hindrance affects both medical and non-medical applications, but higher effect is for the non-medical applications due to the wider use outside of a controlled environment. The effect on non-medical applications is also significant as the possibilities for the use of the EEG-based BCI applications in the non-medical domain is relatively wider and number of users is higher in comparison to the applications used in the medical domain.

In the medical domain (in the assistive field), the challenges include low recognition rate for mental commands [158], problems with the signal acquisition equipment reliability and training process [117]. The character of the brain signals and the amplitude varies between persons, which makes it more difficult to develop BCI applications suitable for all patients [6]. The work on separating specific EEG signals from other signals [234] and the hindrances concerning the aspect of cross-subject classification [235] and accuracy of interpreting the commands [236] is ongoing. The authors agree that one of the important aspects is also the low throughput of information which may be a limiting factor in some applications [117,158]. The challenge of the low accuracy of the system has also been experienced in the field of monitoring emotional responses [161]. In the rehabilitation field, the complexity of the system setup and high cost of the devices has been noted [163]. In neurorehabilitation, it has been highlighted that the success of the therapeutic methods is hard to measure and the repetitiveness that is needed from the subject during the therapy could be demotivating [106]. Further research needs to be conducted to overcome the repetitions needed by the patient during the use of the BCI and possibilities to overcome time-consuming calibration for the BCI users [237].

Concerning BCI applications in the non-medical domain, Wei et al. have highlighted that in order to transition laboratory-oriented BCI applications into real-world environments, the convenience to use and long-term wearing comfort for the devices needs to be improved [193]. There have been alternative devices created for this reason, to increase for example the user-friendliness of the devices meant to measure drowsiness at work [14]. During detection of human emotions, the hinderance could be the ambiguity of human emotions and the complexity of EEG signals [203]. There has also been works on the closed-loop interactions of human emotions with emotional stimuli for example in the case of music interface which complicates the system setup further [77]. In the field of entertainment, it has been emphasized that the seamless interaction between user and the device is of utmost importance and a crucial concern [110].

The challenges hindering the development of BCI applications in both medical and non-medical settings involve poor portability of the hardware, low user-friendliness of the device, low signal quality and individual differences among persons that result in difficulties interpreting the EEG signals across users [6,193,238,239]. The accuracy of EEG pattern decoding is one of the key aspects for developing reliable BCI applications [240]. In current development of BCI applications the variability of EEG signals received from one subject and inter-subject variability has remained one of the most important obstacles [241] and the designing of subject-independent BCI systems has remained a challenge [242].

The use of current subject-dependent applications is time consuming due to training and especially inconvenient for people with mental disabilities [243]. Therefore, in addition to the BCI application functioning for one specific person, it would be important to develop subject-independent BCI systems that can be used by various users without previous training [241]. The challenge in developing subject-independent BCI applications is to overcome person-dependent scenarios where the training and sets come from the same person and would require high-performance person-independent classification [238]. Overcoming the challenge would enable efficient use of subject-independent system in turn enabling wider implementation of the BCI applications.

Both medical and non-medical domains are affected by potential ethical issues in the EEG-based BCI applications filed of research. It would need to be determined for example who would be liable in case of any accidents during the use of BCI applications or could the applications affect, for example, user’s mood and therefore affect user’s decision making in a broader sense [2]. It has been shown that under certain circumstances it would be possible to probe subliminally private information from the users using EEG-based BCI devices [83]. In case the ethical and security aspects of the BCI applications are not controlled, an especially wider spread of non-medical devices could be used to exploit user emotions to push targeted marketing and political agendas [2]. These aspects would need to be considered and the users would need to be notified about the possible risks and responsibilities. Ethical standards would need to be established in order to guide the general development of BCI technology and prevent ethical issues preemptively [2].

### 6.2. Future Possibilities

It has been suggested that in order to make the EEG-based BCI applications more accurate and efficient hybrid BCI systems should be developed that combine BCI systems with another BCI or other kinds of interfaces [2]. In addition to the use of only EEG for obtaining the biological signals, other methods could be used to support the strength and quality of the signals such as fNIRS or fMRI [6]. Such other physiological measures could also be heart rate or eye movements [1].

Increasing attention has been paid to fNIRS due to the advantages such as non-invasiveness, user safety, affordability and portability, but fNIRS signals are highly subject-specific and have low test-retest reliability [244]. Barrios et al. [245] have also suggested that combining fMRI with EEG could overcome significant limitations that are present in the use of EEG alone. Padfield et al. [2] have suggested to combine as well different approaches using EEG, such as motor-imagery system together with steady-state visually evoked potential as a training aid. In addition, combining different methods such as fNIRS with motor-imagery EEG or motor-imagery EEG with sensory interface has been proposed by Padfield et al. [2].

#### 6.2.1. Possibilities in Medical Domain

In the medical domain, the BCI could be used to control a variety of assistive robotic devices. EEG-based BCI applications have been developed for controlling wheelchairs [111,200], and research has been conducted on the development of robotic limbs [158]. Recent studies have shown additional success for the development of EEG-based BCI applications for controlling robotic devices [246,247]. Further research could be pursued in order to develop humanoid robots and drones for the support of daily activities for the patients [6].

It has been further suggested to use virtual reality in neuro rehabilitation, where motor-imagery BCI virtual reality systems could be used for real time applications for stroke rehabilitation, to increase motivation of the patient during the rehabilitation process [106]. The monitoring of EEG could be used to enhance speech therapy sessions for people who stutter, providing real-time visualization of the brain and insights concerning the brain activity during the sessions [43]. Jeunet et al. [11] have suggested BCI neurofeedback for the reduction of hypokinetic activity in case of a stroke and reducing hyperkinetic activity in case of attention deficit hyperactivity disorder (ADHD).

#### 6.2.2. Possibilities in Non-Medical Domain

In the non-medical domain, there is a high potential for the EEG-based BCI applications monitoring cognitive load, attention, drowsiness and other aspects of the mind. Monitoring cognitive load has been studied by Friedman et al. for intelligence tests, but measuring cognitive load could also be used to better design and conduct e-learning, psychometric exams, military training and other trainings [84].

Several studies have been published on monitoring attention via EEG-based BCI applications [3,150,171]. The level of attention is essential, both in the learning process and during tasks with high responsibility. Sethi et al. [171] have developed EEG-based attention feedback to improve focus in e-learning, but the principles could also be applied for drivers to test their reflexes and attentiveness and for driving instructors to assess the capability of the drivers. The monitoring of attention level could also be used to correlate stress with attention level and creativity with attention level. According to Sethi et al. [171] for example, optimal stress level can boost attention, and with the help of monitoring, attention optimal stress level could be determined, i.e., when the attention level for the individual is the highest.

EEG-based BCI applications could be further developed for wider authentication of persons in addition to currently available methods [37]. It has been suggested that EEG data could also be used for deceit identification. As polygraphy test is not fully reliable and the results could be altered in case of specific practice and training by the subject. EEG data could be an important alternative for deceit identification in the future [74]. Punsawad et al. [161] have suggested that the monitoring of human emotions via EEG-based BCI application could be applied in neuromarketing for product branding and advertising slogan design.

Entertainment is an ever-developing field and includes, for example, gaming. The availability and user friendliness of the games using EEG-based BCI has been increasing over time [110]. With further development of the technology and integration with currently available technologies there are numerous possibilities for the use of EEG-based BCI for games for both entertainment and serious games for training purposes [8].

The main reasons for the higher pace of development in the non-medical domain are due to the high potential of the domain for a wider range of users when compared to the medical domain. While development in medical domain is focusing on specific patient groups the EEG-based BCI applications in non-medical domain could be used by the general public, for example for smart home control, entertainment or gaming. The range of use for the devices in the non-medical domain is continuously broadening as new possibilities emerge for the use of the devices in new situations such as deceit identification test [74] or authentication based on emotionally significant images [166].

EEG-based BCI applications have a wide variety of applications in both medical and non-medical domains, with even higher potential recently in the non-medical domain due to a high number of potential users around the world. With the development of the technology, reduction in cost and increase in comfort of use the EEG-based BCI applications can gain increasing attention in the non-medical domain.

#### 6.2.3. Safety and User-Friendliness

During the daily use of EEG-based BCI applications, the safety of use is one of the most important aspects to focus on. In case of synchronous BCI, the controlling of the BCI is divided into time windows when the commands by the user could be given and the transfer of commands from the user to the device is well defined. For the user, asynchronous BCI applications would be preferred, as in case of asynchronous applications the commands could be given to the device at any time, independent of specific time windows. From the safety perspective, it is important to define, in case of asynchronous devices, when the user actually wanted to give command and when the user was thinking other thoughts not related to the use of the device. For this reason, the concept of “brain switch” would need to be further developed in practice, enabling the user to mentally disconnect from the device when the user is not intending to use the device [6,131].

Further possibilities for the EEG-based BCI applications include new, more convenient methods for obtaining EEG signals. Wei et al. [193] have suggested measuring EEG from non-hair-bearing scalp areas for the further ease of use during long-term usage of the EEG-based BCI devices in everyday situations. It has also been shown that in some cases it would be sufficient to measure EEG only near the ear that could make the use of EEG-based BCI applications more convenient [113]. In order to make the use of BCI applications more efficient and further integrate the possibilities into our daily tasks, BCIs could be integrated with augmented reality which would create new dimensions of user experience and practicality [15].

## 7. Analysis/Synthesis

In the current review, both articles and conference proceedings have been included. The inclusion of both these types in the analysis gives better representation of the ideas in the field and helps reduce possible publication bias.

Although initially the EEG-based BCI applications were mainly developed for medical purposes to help patients and support in daily activities, the focus has been moving from the medical domain to non-medical applications. The shift in focus does not reduce the importance of these applications in the medical domain but rather shows the wider potential of EEG-based BCI applications and opens new doors for applying the possibilities mode broadly.

As per region, 111 publications (55%) have been contributed by Asia being firmly in the lead concerning number of studies published. From Asia most of the publications per country have been contributed by China. This is a relevant finding as China has increasing influence in general in high technology sector and EEG-based BCI could be one important field of research resulting in a high variety of high technology applications designed for many different fields of life, both medical and non-medical. The high potential of EEG-based BCI applications and diverse possibilities for use could support the future economic strength of the countries and regions investing in the research and development in the field.

The reasons of the general popularity of Emotiv EPOC (Emotiv, San Francisco, CA, USA) EEG device are its relatively low cost, sufficient number of EEG channels and it being considered easy to use. Emotiv EPOC (Emotiv, San Francisco, CA, USA) and MindWave (NeuroSky, San Jose, CA, USA) are considered to be low-cost consumer EEG devices whereas Quik-Cap (Compumedics Neuroscan, Charlotte, NC, USA) is higher in cost. Emotiv EPOC (Emotiv, San Francisco, CA, USA) EEG device has 14 channels and MindWave (NeuroSky, San Jose, CA, USA) 1 channel, whereas the Quik-Cap (Compumedics Neuroscan, Charlotte, NC, USA) EEG device uses 32 channels. MindWave (NeuroSky, San Jose, CA, USA) EEG device has limitations in use due to the limited number of EEG channels, but the low cost and ease of use make the device popular. The device could be applied in specific applications that do not require higher number of EEG channels. The Quik-Cap (Compumedics Neuroscan, Charlotte, NC, USA) device on the other hand is more expensive but has the advantage of higher number of EEG channels. The final decision concerning the use of a specific EEG device depends on the type of EEG-based BCI application, determining the need for specific number of EEG channels. The decision also depends on the cost planned for the study and the end users.

When planning the selection of the EEG device and number of channels, it is important to consider the end users for the EEG-based BCI application, which determines the technical requirements and possible overall cost of the device. A smaller number of carefully selected electrode positions would also make the device more user friendly and support the performance of the system.

There is a general trend noted in the overall use of various techniques. As in case of motor-imagery, auditory evoked potential and vibrotactile evoked potential technique the main application is in the medical domain, but the majority and larger variety of techniques are applied in the non-medical domain. As seen from the results of the current review, a highly diverse selection of techniques has been applied in the non-medical field supporting further the development of diverse applications and supporting the high potential of non-medical applications among the EEG-based BCI applications.

In the current review, a trend of high prevalence of using power spectral density and Fourier transform for feature extraction has been noted. The overall results correlate with previous studies in the field, highlighting the importance of these methods for feature extraction and emphasizing the need to apply different types of feature extraction methods depending on the application under study. The results of the current review also show the importance of linear discriminant analysis and support vector machine for classification in correlation with previous studies in the field. The choice for classification algorithm depends on the type of brain signal being recorded and the type of application that is being controlled.

With the widespread use of the devices, the safety on an individual and community level would need to be further analyzed. Nowadays, it has been unfortunately common that due to security breaches malicious software have infiltrated computer networks even with a high security level. This needs to be taken into consideration, especially when considering the sensitivity of the biomedical information obtained from the BCI device and also the possibility to alter the brain signals for example via neurofeedback. As the field of EEG-based BCI is fast developing, the ethical aspects would need to be analyzed and safeguarded in parallel with the development of technology.

The BCI applications are developing rapidly and therefore it is important to have an up-to-date overview on the EEG-based BCI applications together with possible challenges and the way forward. The significance of the current review is to provide an overview of the current work conducted in the field of EEG-based BCI applications, along with the challenges and future possibilities.

## Figures and Tables

**Table 1:** Categorization of BCIs and corresponding techniques used to obtain EEG data according to Al-Nafjan et al. [5] and Abiri et al. [1].

Category	Technique Used to Obtain EEG Data	Description
Active	Motor-imagery	Imagining the movement of a specific body part for example hands, feet or tongue. The intent will affect the brain activity and could be detected from the EEG. The imagination activates the brain areas that are responsible for generating the actual movement.
	Visual evoked potential	The brain activity is affected by external visual stimulation and the corresponding altered EEG activity is registered. For example, in case of steady-state visual evoked potential (SSVEP) there are different visual stimuli flickering at different frequencies and depending on the direction of the gaze of the subject the EEG pattern will be consistent with the specific flickering rate.
	Auditory evoked potential	Auditory stimulation is generated and depending on the focus of the subject corresponding EEG activity is registered.
	Vibrotactile evoked potential	Physical vibrations at different frequencies are generated for example on the hands and feet of the subject. Depending on the focus of the subject, a corresponding EEG pattern to the specific physical vibration is registered and could be used to control some external device.
	Imagined speech	Imagination of words or sentences that are recognized from EEG.
	Error-related potential	The error-related potential is generated when there is a mismatch between the subject’s intentions and response from the BCI application. The technique can be used to correct tasks given by the subject. For example, when the subject is controlling the cursor, but the cursor is moving in the wrong direction, an error-related potential is generated, and the course of the cursor can be corrected.
Passive	Analyzing EEG spectral changes	Systems wherein brain signals yield outputs without any voluntary control. For example, monitoring drowsiness, attention, mental workload, emotions, concentration and other states of the mind.

**Table 2:** Additional different categorization of the BCIs in the literature by Pasqualotto et al. [23], Machado et al. [26], Padfield et al. [2] and Nicolas-Alonso and Gomez-Gil [27].

Author	BCI Categorization	Description
Pasqualotto et al. [23] Machado et al. [26]	Dependent	Dependent on muscles and peripheral nerves. For example, in case of visual evoked potential (VEP), gaze is directed by muscles to focus on different visual stimuli.
	Independent	Muscle movement is not needed to control BCI. For example, in case of P300 response is detected from EEG and analyzed.
Padfield et al. [2]	Evoked	Also named as exogenous. Some type of external stimulation is required such as visual, auditory, or sensory. Can be further divided to evoked potentials and event-related potentials. In case of evoked potentials, changes in EEG can be detected due to responses to external stimuli. In case of event-related potentials, the changes in EEG are caused by sensory or cognitive events.
	Spontaneous	Also named as endogenous. External stimulation is not required. For example, motor-imagery technique, where subjects imagine movement of a limb and there is no additional input from external stimuli.
Nicolas-Alonso and Gomez-Gil [27]	Synchronous	The BCI analyzes signals during certain time windows, and the subject is able to give commands after fixed time intervals.
	Asynchronous	The brain waves of the subject are analyzed constantly, and the subject can give commands whenever the subject wants. Asynchronous BCI gives the subject more possibilities and flexibility concerning controlling the BCI.

## Appendix A. Search Terms Used

The search was conducted in the databases PubMed, Scopus and Web of Science using basic search settings with the below-described search terms relevant for current review. The search was conducted in PubMed database on 20 October 2019 with the search term (“electroencephalography based” OR “EEG based”) AND (“brain computer interface” OR “BCI” OR “brain machine interface” OR “BMI”) AND (“application” OR “applications”). The search resulted in 185 publications that were then included for further screening.

The search in the Web of Science database was conducted on 28 October 2019 with the search term (“electroencephalography based” OR “EEG based”) AND (“brain computer interface” OR “BCI” OR “brain machine interface” OR “BMI”) AND (“application” OR “applications”) and the search in Web of Science database resulted in 451 publications.

The search in the Scopus database was conducted on 30 October 2019 using search term (“electroencephalography based” OR “EEG based”) AND (“brain computer interface” OR “BCI” OR “brain machine interface” OR “BMI”) AND (“application” OR “applications”) and 569 publications were identified through the search for inclusion to further screening.

## Appendix B

**Table A1:** Listing of Additional Publications from the Period of 31 October 2019 Until 26 June 2021.

Author	Year Published	Title
Choi et al.	2019	A multi-day and multi-band dataset for a steady-state visual-evoked potential-based brain–computer interface
Ganorkar and Raut	2019	Comparative analysis of mother wavelet selection for eeg signal application to motor imagery-based brain–computer interface
Hekmatmanesh et al.	2019	Combination of discrete wavelet packet transform with detrended fluctuation analysis using customized mother wavelet with the aim of an imagery-motor control interface for an exoskeleton
Khan et al.	2019	Multiclass EEG motor-imagery classification with sub-band common spatial patterns
Khoshnevis and Ghorshi	2019	Recovery of event-related potential signals using compressive sensing and kronecker technique
Liu et al.	2019	Fully Passive Flexible Wireless Neural Recorder for the Acquisition of Neuropotentials from a Rat Model
Mebarkia and Reffad	2019	Multi optimized SVM classifiers for motor imagery left and right hand movement identification
Nagabushan et al.	2019	A comparative study of motor imagery-based BCI classifiers on EEG and iEEG data
Onay and Kose	2019	Assessment of CSP-based two-stage channel selection approach and local transformation-based feature extraction for classification of motor imagery/movement EEG data
Oralhan	2019	2 Stages-region-based P300 Speller in Brain–Computer Interface
Saikia and Paul	2019	EEG signal processing and its classification for rehabilitation device control
Taran and Bajaj	2019	Motor imagery tasks-based EEG signals classification using tunable-Q wavelet transform
Wu et al.	2019	A Parallel Multiscale Filter Bank Convolutional Neural Networks for Motor Imagery EEG Classification
Yao and Shoaran	2019	Enhanced Classification of Individual Finger Movements with ECoG
Yu Chen and Mehmood	2019	A critical review on state-of-the-art EEG-based emotion datasets
Zhang et al.	2019	A Graph-Based Hierarchical Attention Model for Movement Intention Detection from EEG Signals
Zhang et al.	2019	Deep Learning Decoding of Mental State in Non-invasive Brain Computer Interface
Aggarwal and Chugh	2020	A decade of EEG Analysis: Prospects & Challenges in Biometric System
Alakus and Turkoglu	2020	Emotion recognition with deep learning using GAMEEMO data set
Alhakeem et al.	2020	Wheelchair Free Hands Navigation Using Robust DWT-AR Features Extraction Method with Muscle Brain Signals
Ali et al.	2020	Classification of Motor Imagery Task by Using Novel Ensemble Pruning Approach
Al-Nafjan et al.	2020	Lightweight Building of an Electroencephalogram-Based Emotion Detection System
Andrade et al.	2020	An EEG Brain–Computer Interface to Classify Motor Imagery Signals
Angrisani et al.	2020	Instrumentation for motor imagery-based brain computer interfaces relying on dry electrodes: A functional analysis
Araki et al.	2020	Wireless Monitoring Using a Stretchable and Transparent Sensor Sheet Containing Metal Nanowires
Arico et al.	2020	Brain–Computer Interfaces: Toward a Daily Life Employment
Aroudi and Doclo	2020	Cognitive-Driven Binaural Beamforming Using EEG-Based Auditory Attention Decoding
Bablani et al.	2020	A multi stage EEG data classification using k-means and feed forward neural network
Bigirimana et al.	2020	Emotion-Inducing Imagery Versus Motor Imagery for a Brain–Computer Interface
Borra et al.	2020	Interpretable and lightweight convolutional neural network for EEG decoding: Application to movement execution and imagination
Borra et al.	2020	Convolutional Neural Network for a P300 Brain–Computer Interface to Improve Social Attention in Autistic Spectrum Disorder
Cao and Grover	2020	STIMULUS: Noninvasive Dynamic Patterns of Neurostimulation Using Spatio-Temporal Interference
Castro et al.	2020	Development of a Deep Learning-Based Brain–Computer Interface for Visual Imagery Recognition
Cha et al.	2020	Prediction of individual user’s dynamic ranges of EEG features from resting-state EEG data for evaluating their suitability for passive brain–computer interface applications
Chamola et al.	2020	Brain–computer interface-based humanoid control: A review
Chen et al.	2020	EEG-based biometric identification with convolutional neural network
Chen et al.	2020	Emotion recognition from spatiotemporal EEG representations with hybrid convolutional recurrent neural networks via wearable multi-channel headset
Cheng et al.	2020	Motion Imagery-BCI Based on EEG and Eye Movement Data Fusion
Cho et al.	2020	A Novel Approach to Classify Natural Grasp Actions by Estimating Muscle Activity Patterns from EEG Signals
Cho et al.	2020	Decoding of Grasp Motions from EEG Signals Based on a Novel Data Augmentation Strategy
Cortez et al.	2020	Improving Speller BCI performance using a cluster-based under-sampling method
Cortez et al.	2020	Under-sampling and Classification of P300 Single-Trials using Self-Organized Maps and Deep Neural Networks for a Speller BCI
Cozza et al.	2020	Dimension Reduction Techniques in a Brain–Computer Interface Application
Cudlenco et al.	2020	Reading into the mind’s eye: Boosting automatic visual recognition with EEG signals
de Melo et al.	2020	EEG Analysis in Coincident Timing Task Towards Motor Rehabilitation
Delvigne et al.	2020	Attention Estimation in Virtual Reality with EEG-based Image Regression
Deng et al.	2020	Self-adaptive shared control with brain state evaluation network for human-wheelchair cooperation
Deng et al.	2020	A Bayesian Shared Control Approach for Wheelchair Robot with Brain Machine Interface
Dimitrov et al.	2020	Increasing the Classification Accuracy of EEG-based Brain–computer Interface Signals
Dutta and Nandy	2020	An extensive analysis on deep neural architecture for classification of subject-independent cognitive states
Dutta et al.	2020	Development of a BCI-based gaming application to enhance cognitive control in psychiatric disorders
Elessawy et al.	2020	A long short-term memory autoencoder approach for EEG motor imagery classification
Elkafrawy et al.	2020	Proposed model for thought-based animation based on classifying EEG signals using estimated parameters and multi-SVM
Fathima and Kore	2020	Enhanced Differential Evolution-Based EEG Channel Selection
Feng et al.	2020	Decoding of voluntary and involuntary upper-limb motor imagery based on graph fourier transform and cross-frequency coupling coefficients
Filipp et al.	2020	Application of brain–computer interfaces in assistive technologies
Fontanillo et al.	2020	Beyond Technologies of Electroencephalography-Based Brain–Computer Interfaces: A Systematic Review From Commercial and Ethical Aspects
Gembler et al.	2020	Five Shades of Grey: Exploring Quintary m-Sequences for More User-Friendly c-VEP-Based BCIs
Ghosh et al.	2020	Bi-directional Long Short-Term Memory model to analyze psychological effects on gamers
Gorriz et al.	2020	Artificial intelligence within the interplay between natural and artificial computation: Advances in data science, trends and applications
Grissmann et al.	2020	Context Sensitivity of EEG-Based Workload Classification Under Different Affective Valence
Gu et al.	2020	The effects of varying levels of mental workload on motor imagery-based brain computer interface
Gubert et al.	2020	The performance impact of data augmentation in CSP-based motor-imagery systems for BCI applications
Gurve et al.	2020	Trends in Compressive Sensing for EEG Signal Processing Applications
Haira et al.	2020	A comparison of ECG and EEG metrics for in-flight monitoring of helicopter pilot workload
Hernandez-Cuevas et al.	2020	Neurophysiological Closed-Loop Control for Competitive Multi-brain Robot Interaction
Hussain and Park	2020	HealthSOS: Real-Time Health Monitoring System for Stroke Prognostics
Idowu et al.	2020	Efficient Classification of Motor Imagery using Particle Swarm Optimization-based Neural Network for IoT Applications
Ieracitano et al.	2020	A novel multi-modal machine learning-based approach for automatic classification of EEG recordings in dementia
Jeng et al.	2020	Low-Dimensional Subject Representation-based Transfer Learning in EEG Decoding
Jin et al.	2020	EEG classification using sparse Bayesian extreme learning machine for brain–computer interface
Kalafatovich et al.	2020	Decoding Visual Recognition of Objects from EEG Signals based on Attention-Driven Convolutional Neural Network
Kang et al.	2020	EEG-Based Prediction of Successful Memory Formation During Vocabulary Learning
Kaongoen and Jo	2020	An Ear-EEG-based Brain–Computer Interface using Concentration Level for Control
Kaur et al.	2020	A study of EEG for enterprise multimedia security
Khan et al.	2020	High performance multi-class motor imagery EEG classification
Kouddad et al.	2020	Indexing and Image Search by the Content According to the Biological Base of the Cognitive Processing of Information using a Neural Sensor
Kurapa et al.	2020	A Hybrid Approach for Extracting EMG signals by Filtering EEG Data for IoT Applications for Immobile Persons
Kuzovkin et al.	2020	Mental state space visualization for interactive modeling of personalized BCI control strategies
Kwon et al.	2020	Decoding of Intuitive Visual Motion Imagery Using Convolutional Neural Network under 3D-BCI Training Environment
Landau et al.	2020	Mind Your Mind: EEG-Based Brain–Computer Interfaces and Their Security in Cyber Space
Lee et al.	2020	Complex Motor Imagery-based Brain–Computer Interface System: A Comparison between Different Classifiers
Lee et al.	2020	Classification of Upper Limb Movements Using Convolutional Neural Network with 3D Inception Block
Leon et al.	2020	Deep learning for EEG-based Motor Imagery classification: Accuracy-cost trade-off
Li et al.	2020	Enhancing BCI-Based Emotion Recognition Using an Improved Particle Swarm Optimization for Feature Selection
Liang et al.	2020	EEG-Based EMG Estimation of Shoulder Joint for the Power Augmentation System of Upper Limbs
Lin et al.	2020	A Multi-Scale Activity Transition Network for Data Translation in EEG Signals Decoding
Luo et al.	2020	EEG Signal Reconstruction Using a Generative Adversarial Network With Wasserstein Distance and Temporal-Spatial-Frequency Loss
Luo et al.	2020	Estimation of Motor Imagination Based on Consumer-Grade EEG Device
Ma et al.	2020	Online learning using projections onto shrinkage closed balls for adaptive brain computer interface
Mattia et al.	2020	The Promotoer, a brain–computer interface-assisted intervention to promote upper limb functional motor recovery after stroke: a study protocol for a randomized controlled trial to test early and long-term efficacy and to identify determinants of response
Miao et al.	2020	Spatial-Frequency Feature Learning and Classification of Motor Imagery EEG Based on Deep Convolution Neural Network
Min and Cai	2020	Driver Fatigue Detection Based on Multi-scale Wavelet Log Energy Entropy of Frontal EEG
Mishra et al.	2020	Effect of hand grip actions on object recognition process: a machine learning-based approach for improved motor rehabilitation
Mondini et al.	2020	Continuous low-frequency EEG decoding of arm movement for closed-loop, natural control of a robotic arm
Nakagome et al.	2020	An empirical comparison of neural networks and machine learning algorithms for EEG gait decoding
Netzer et al.	2020	Real-time EEG classification via coresets for BCI applications
Nisar et al.	2020	Reducing Sensors in Mental Imagery-Based Cognitive Task for Brain Computer Interface
Pa Aung and New	2020	Regions of Interest (ROI) Analysis for Upper Limbs EEG Neuroimaging Schemes
Padmavathy et al.	2020	A novel deep learning classifier and genetic algorithm-based feature selection for hybrid EEG-FNIRS brain–computer interface
Paek et al.	2020	Towards a Portable Magnetoencephalography-Based Brain Computer Interface with Optically-Pumped Magnetometers
Pan et al.	2020	Prognosis for patients with cognitive motor dissociation identified by brain–computer interface
Pan et al.	2020	EEG-Based Emotion Recognition Using Logistic Regression with Gaussian Kernel and Laplacian Prior and Investigation of Critical Frequency Bands
Parikh and George	2020	Quadcopter Control in Three-Dimensional Space Using SSVEP and Motor Imagery-Based Brain–Computer Interface
Petukhov et al.	2020	Being present in a real or virtual world: A EEG study
Philip and George	2020	Visual P300 Mind-Speller Brain–Computer Interfaces: A Walk Through the Recent Developments With Special Focus on Classification Algorithms
Qin et al.	2020	Smart Home Control for Disabled Using Brain Computer Interface
Rakshit et al.	2020	A Hybrid Brain–Computer Interface for Closed-Loop Position Control of a Robot Arm
Rashid et al.	2020	Current Status, Challenges, and Possible Solutions of EEG-Based Brain–Computer Interface: A Comprehensive Review
Rashid et al.	2020	Five-Class SSVEP Response Detection using Common-Spatial Pattern (CSP)-SVM Approach
Riyad et al.	2020	Incep-eegnet: A convnet for motor imagery decoding
Roy et al.	2020	A hybrid classifier combination for home automation using EEG signals
Sadiq et al.	2020	Identification of motor and mental imagery EEG in two and multiclass subject-dependent tasks using successive decomposition index
Sahu et al.	2020	EEG signal analysis and classification on P300 speller-based BCI performance in ALS patients
Schembri et al.	2020	The Effect That Auditory Distractions Have on a Visual P300 Speller While Utilizing Low Cost Off-the-Shelf Equipment
Schneider et al.	2020	Real-time EEG Feedback on Alpha Power Lateralization Leads to Behavioral Improvements in a Covert Attention Task
Shao et al.	2020	EEG-Controlled Wall-Crawling Cleaning Robot Using SSVEP-Based Brain–Computer Interface
She et al.	2020	Multi-class motor imagery EEG classification using collaborative representation-based semi-supervised extreme learning machine
Shi et al.	2020	Feature Extraction of Brain–Computer Interface Electroencephalogram Based on Motor Imagery
Siddharth and Trivedi	2020	On assessing driver awareness of situational criticalities: Multi-modal bio-sensing and vision-based analysis, evaluations, and insights
Singh and Singh	2020	Realising transfer learning through convolutional neural network and support vector machine for mental task classification
Song et al.	2020	A Practical EEG-Based Human-Machine Interface to Online Control an Upper-Limb Assist Robot
Suma et al.	2020	Spatial-temporal aspects of continuous EEG-based neurorobotic control
Sun et al.	2020	Multimodal affective state assessment using fNIRS+ EEG and spontaneous facial expression
Talukdar et al.	2020	Adaptive feature extraction in EEG-based motor imagery BCI: tracking mental fatigue
Tan et al.	2020	Spiking Neural Networks: Background, Recent Development and the NeuCube Architecture
Tao	2020	Classification-Oriented Fuzzy-Rough Feature Selection for the EEG-Based Brain Computer Interfaces
Tiwari et al.	2020	Machine Learning approach for the classification of EEG signals of multiple imagery tasks
Torkamani-Azar et al.	2020	Prediction of Motor Imagery Performance based on Pre-Trial Spatio-Spectral Alertness Features
Torres et al.	2020	EEG-Based BCI Emotion Recognition: A Survey
Tzdaka et al.	2020	Assessing the Relevance of Neurophysiological Patterns to Predict Motor Imagery-based BCI Users’ Performance
Vigue-Guix et al.	2020	Can the occipital alpha-phase speed up visual detection through a real-time EEG-based brain–computer interface (BCI)?
Wafeek et al.	2020	A Novel EEG Classification Technique Based on Particle Swarm Optimization for Hand and Finger Movements
Wang et al.	2020	Enhancing gesture decoding performance using signals from posterior parietal cortex: a stereo-electroencephalograhy (SEEG) study
Wang et al.	2020	P300 Recognition Based on Ensemble of SVMs
William et al.	2020	ERP Template Matching for EEG Single Trial Classification
Wolpaw et al.	2020	Brain–computer interfaces: Definitions and principles
Xu et al.	2020	Implementing Over 100 Command Codes for a High-Speed Hybrid Brain–Computer Interface Using Concurrent P300 and SSVEP Features
Xu et al.	2020	Motor Imagery-Based Continuous Teleoperation Robot Control with Tactile Feedback
Xu et al.	2020	Two-level multi-domain feature extraction on sparse representation for motor imagery classification
Yan et al.	2020	An improve d common spatial pattern combine d with channel-selection strategy for electroencephalography-based emotion recognition
Yang et al.	2020	MI3DNet: A Compact CNN for Motor Imagery EEG Classification with Visualizable Dense Layer Parameters
Yao et al.	2020	Information-preserving feature filter for short-term EEG signals
Yi	2020	Efficient machine learning algorithm for electroencephalogram modeling in brain–computer interfaces
Zeng et al.	2020	InstanceEasyTL: An Improved Transfer-Learning Method for EEG-Based Cross-Subject Fatigue Detection
Zhang et al.	2020	Pain Control by Co-adaptive Learning in a Brain–Machine Interface
Zhang et al.	2020	Application of transfer learning in eeg decoding based on brain–computer interfaces: A review
Zhou et al.	2020	A Hybrid Asynchronous Brain–Computer Interface Combining SSVEP and EOG Signals
Zhuang et al.	2020	State-of-the-art non-invasive brain–computer interface for neural rehabilitation: A review
Aldayel et al.	2021	Consumers’ Preference Recognition Based on Brain–Computer Interfaces: Advances, Trends, and Applications
Alhudhaif	2021	An effective classification framework for brain–computer interface system design based on combining of fNIRS and EEG signals
Al-Saegh et al.	2021	Deep learning for motor imagery EEG-based classification: A review
Alzahab et al.	2021	Hybrid deep learning (Hdl)-based brain–computer interface (bci) systems: A systematic review
Asogbon et al.	2021	A linearly extendible multi-artifact removal approach for improved upper extremity EEG-based motor imagery decoding.
Aydarkhanov et al.	2021	Closed-loop EEG study on visual recognition during driving
Belo et al.	2021	EEG-Based Auditory Attention Detection and Its Possible Future Applications for Passive BCI
Benaroch et al.	2021	Long-Term BCI Training of a Tetraplegic User: Adaptive Riemannian Classifiers and User Training
Bhattacharyya et al.	2021	Neuro-feedback system for real-time BCI decision prediction
Cattai et al.	2021	Phase/Amplitude Synchronization of Brain Signals During Motor Imagery BCI Tasks.
Chaudhary et al.	2021	Neuropsychological and neurophysiological aspects of brain–computer interface (BCI) control in paralysis
Chen et al.	2021	EEG-Based Anxious States Classification Using Affective BCI-Based Closed Neurofeedback System
Chen et al.	2021	Implementing a calibration-free SSVEP-based BCI system with 160 targets.
Fu et al.	2021	Recognizing single-trial motor imagery EEG based on interpretable clustering method
Georgiev et al.	2021	Virtual reality for neurorehabilitation and cognitive enhancement
Gu et al.	2021	EEG-based Brain–Computer Interfaces (BCIs): A Survey of Recent Studies on Signal Sensing Technologies and Computational Intelligence Approaches and Their Applications
Guner and Erkmen	2021	A Low-Cost Real-Time BCI Integration for Automated Door Opening System
Gupta et al.	2021	Brain computer interface controlled automatic electric drive for neuro-aid system
Hong et al.	2021	Dynamic Joint Domain Adaptation Network for Motor Imagery Classification
Islam et al.	2021	Auditory Evoked Potential (AEP)-Based Brain–Computer Interface (BCI) Technology: A Short Review
Islam et al.	2021	Probability mapping-based artifact detection and removal from single-channel EEG signals for brain–computer interface applications.
Jeng et al.	2021	Low-Dimensional Subject Representation-Based Transfer Learning in EEG Decoding
Ketu et al.	2021	Hybrid classification model for eye state detection using electroencephalogram signals
Khan et al.	2021	Analysis of Human Gait Using Hybrid EEG-fNIRS-Based BCI System: A Review
Kharchenko et al.	2021	Influence of Signal Preprocessing When Highlighting Steady-State Visual Evoked Potentials Based on a Multivariate Synchronization Index
Kharchenko et al.	2021	Implementation of robot–human control bio-interface when highlighting visual-evoked potentials based on multivariate synchronization index
Kumar et al.	2021	The classification of EEG-based winking signals: a transfer learning and random forest pipeline
Liu et al.	2021	P300 event-related potential detection using one dimensional convolutional capsule networks
Liu et al.	2021	Multiscale space-time-frequency feature-guided multitask learning CNN for motor imagery EEG classification
Liu et al.	2021	A Utility Human Machine Interface Using Low Cost EEG Cap and Eye Tracker
Miladinovic et al.	2021	Effect of power feature covariance shift on BCI spatial-filtering techniques: A comparative study
Mishra et al.	2021	Effect of hand grip actions on object recognition process: a machine learning-based approach for improved motor rehabilitation
Qi et al.	2021	Spatiotemporal-Filtering-Based Channel Selection for Single-Trial EEG Classification
Qi et al.	2021	Wielding and evaluating the removal composition of common artefacts in EEG signals for driving behaviour analysis.
Rammy et al.	2021	Sequence-to-sequence deep neural network with spatio-spectro and temporal features for motor imagery classification
Ravirahul et al.	2021	Mind Wave Controlled Assistive Robot
Reyes et al.	2021	LSTM-based brain–machine interface tool for text generation through eyes blinking detection
Riyad et al.	2021	A novel multi-scale convolutional neural network for motor imagery classification
Rybar et al.	2021	Decoding of semantic categories of imagined concepts of animals and tools in fNIRS
Saga et al.	2021	Elucidation of EEG Characteristics of Fuzzy Reasoning-Based Heuristic BCI and Its Application to Patient With Brain Infarction
Santos et al.	2021	Comparison of LORETA and CSP for Brain–Computer Interface Applications
Shaban et al.	2021	Classification of Lactate Level Using Resting-State EEG Measurements
Shahbakhti et al.	2021	VME-DWT: An Efficient Algorithm for Detection and Elimination of Eye Blink from Short Segments of Single EEG Channel
Shi et al.	2021	A binary harmony search algorithm as channel selection method for motor imagery-based BCI
Somadder et al.	2021	Frequency Domain CSP for Foot Motor Imagery Classification Using SVM for BCI Application
Stival et al.	2021	Connectivity modeling meets machine learning: The next generation of eeg-based brain computer interfaces
Sulaiman et al.	2021	Offline eeg-based dc motor control for wheelchair application
Sun et al.	2021	WLnet: Towards an Approach for Robust Workload Estimation Based on Shallow Neural Networks
Wang et al.	2021	EEG-based auditory attention decoding using speech-level-based segmented computational models
Xu et al.	2021	Review of brain encoding and decoding mechanisms for EEG-based brain–computer interface
Yoo	2021	Electroencephalogram-based neurofeedback training in persons with stroke: A scoping review in occupational therapy
Yu et al.	2021	Cross-correlation-based discriminant criterion for channel selection in motor imagery BCI systems
Zeng et al.	2021	An EEG-Based Transfer Learning Method for Cross-Subject Fatigue Mental State Prediction
Zhang et al.	2021	Improving EEG Decoding via Clustering-Based Multitask Feature Learning
Zhang et al.	2021	EEG-inception: an accurate and robust end-to-end neural network for EEG-based motor imagery classification
Zhang et al.	2021	Tiny noise, big mistakes: adversarial perturbations induce errors in brain–computer interface spellers
Zolfaghari et al.	2021	Using convolution neural networks pattern for classification of motor imagery in bci system
